# Synergism of Antifungal Activity between Mitochondrial Respiration Inhibitors and Kojic Acid

**DOI:** 10.3390/molecules18021564

**Published:** 2013-01-25

**Authors:** Jong H. Kim, Bruce C. Campbell, Kathleen L. Chan, Noreen Mahoney, Ronald P. Haff

**Affiliations:** Plant Mycotoxin Research Unit, Western Regional Research Center, USDA-ARS, 800 Buchanan St., Albany, CA 94710, USA; E-Mails: bruce.campbell@ars.usda.gov (B.C.C.); kathy.chan@ars.usda.gov (K.L.C.); noreen.mahoney@ars.usda.gov (N.M.); ron.haff@ars.usda.gov (R.P.H.)

**Keywords:** kojic acid, *Aspergillus*, *Penicillium*, *Acremonium*, *Scedosporium*, yeast, hydrogen peroxide, mitochondrial respiration inhibitors, chemosensitization

## Abstract

Co-application of certain types of compounds to conventional antimicrobial drugs can enhance the efficacy of the drugs through a process termed chemosensitization. We show that kojic acid (KA), a natural pyrone, is a potent chemosensitizing agent of complex III inhibitors disrupting the mitochondrial respiratory chain in fungi. Addition of KA greatly lowered the minimum inhibitory concentrations of complex III inhibitors tested against certain filamentous fungi. Efficacy of KA synergism in decreasing order was pyraclostrobin > kresoxim-methyl > antimycin A. KA was also found to be a chemosensitizer of cells to hydrogen peroxide (H_2_O_2_), tested as a mimic of reactive oxygen species involved in host defense during infection, against several human fungal pathogens and *Penicillium* strains infecting crops. In comparison, KA-mediated chemosensitization to complex III inhibitors/H_2_O_2_ was undetectable in other types of fungi, including *Aspergillus flavus*, *A. parasiticus*, and *P. griseofulvum*, among others. Of note, KA was found to function as an antioxidant, but not as an antifungal chemosensitizer in yeasts. In summary, KA could serve as an antifungal chemosensitizer to complex III inhibitors or H_2_O_2_ against selected human pathogens or *Penicillium* species. KA-mediated chemosensitization to H_2_O_2_ seemed specific for filamentous fungi. Thus, results indicate strain- and/or drug-specificity exist during KA chemosensitization.

## 1. Introduction

**Figure 1:**
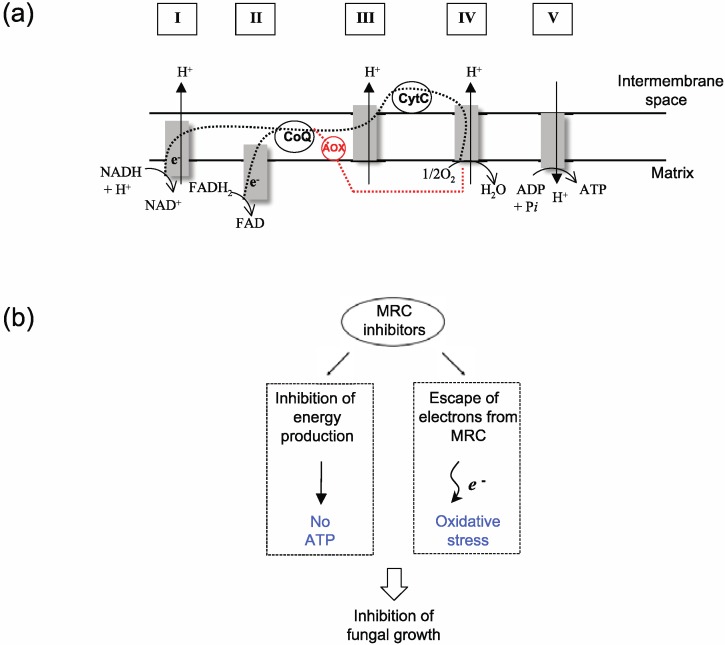
MRC as a target for control of fungal pathogens. (**a**) Schematic representation of MRC (Adapted from [2] and [7]). CoQ, Coenzyme Q; CytC, Cytochrome C; e^−^, Electrons; AOX, Alternative oxidase; Dashed lines (black), Normal route for electron flow; Dashed lines (red), Alternative route for electron flow; I to V, components/complexes of MRC. (**b**) Mechanism of antifungal action of MRC inhibitors.

The mitochondrial respiratory chain (MRC) can serve as a valuable molecular target for control of fungal pathogens (Figure 1a). Chemical inhibitors of MRC, such as antimycin A (AntA) or strobilurins (e.g., Pyraclostrobin (PCS), Kresoxim-methyl (Kre-Me), mucidin, *etc*.), interfere with cellular energy (e.g., ATP) production in fungi [1,2], weakening fungal viability. Coinciding with this interference is an abnormal leakage of electrons from MRC. The escaped electrons can cause oxidative damage to vital components in fungal cells, such as chromosomes, lipid membranes and proteins, resulting in apoptosis or necrosis [1,2] (see Figure 1b for scheme). The antioxidant system in fungi, e.g., glutaredoxins, cytosolic or mitochondrial superoxide dismutases (Cu, Zn- or Mn-SOD), glutathione reductase, plays a protective role in such cases, maintaining cellular homeostasis/integrity from toxic oxidative species [3,4]. Fungi can also overcome the toxicity of MRC inhibitors by expressing alternative oxidase (AOX) (Figure 1a), rendering the completion of electron flow *via* MRC [5,6]. AOX is insensitive to MRC inhibitors [5,6].

With respect to other targets of conventional antifungal drugs already identified (e.g., cell wall/membrane integrity pathway, cell division, signal transduction, and macromolecular synthesis, *etc*.) [8], MRC is a relatively unexploited target in human fungal pathogens. However, the MRC has been actively used as a drug target for control of malarial parasites, e.g., *Plasmodium*. For example, the antimalarial drug atovaquone disrupts the mitochondrial electron transport as well as the inner mitochondrial membrane potential (ΔΨ_m_) in parasites [9]. Atovaquone is also used to treat fungal infections such as *Pneumocystis jirovecii* (pneumonia) [10].

Co-application of certain types of compounds with commercial antimicrobial drugs can increase the effectiveness of drugs through a mechanism termed “chemosensitization” [11,12,13,14]. For example, a prior study showed that the 4-methoxy-2,3,6-trimethylbenzensulfonyl-substituted D-octapeptide chemosensitized cells to the antifungal drug fluconazole (FLC), countering FLC resistance of clinical isolates of *Candida* pathogens, and of strains of the model yeast *Saccharomyces cerevisiae* overexpressing multidrug efflux pumps/drug transporter or a lanosterol 14α-demethylase (Erg11p, molecular target of FLC) [11]. Similarly, in bacterial pathogens, application of sub-inhibitory concentrations of squalamine enhanced the antibiotic susceptibility of various Gram-negative bacteria, in both antibiotic-resistant and susceptible strains [12]. Squalamine is thought to modify membrane integrity by increasing permeability of drugs [12].

Meanwhile, co-application of proguanil, which modulates mitochondria in protozoan parasites, resulted in an increased antimalarial activity of atovaquone [15]. Of note is that proguanil-based chemosensitization was specific for atovaquone, *i.e*., proguanil did not enhance the activities of other MRC inhibitors, such as myxothiazole or AntA [15]. Results indicate “drug-chemosensitizer specificity” exists in the process. Collectively, these studies showed that chemosensitization could ultimately lead to lowering dosages of conventional drugs necessary for effective control of pathogens. It would also lead to preventing development of pathogen resistance to conventional drugs [16].

**Figure 2:**
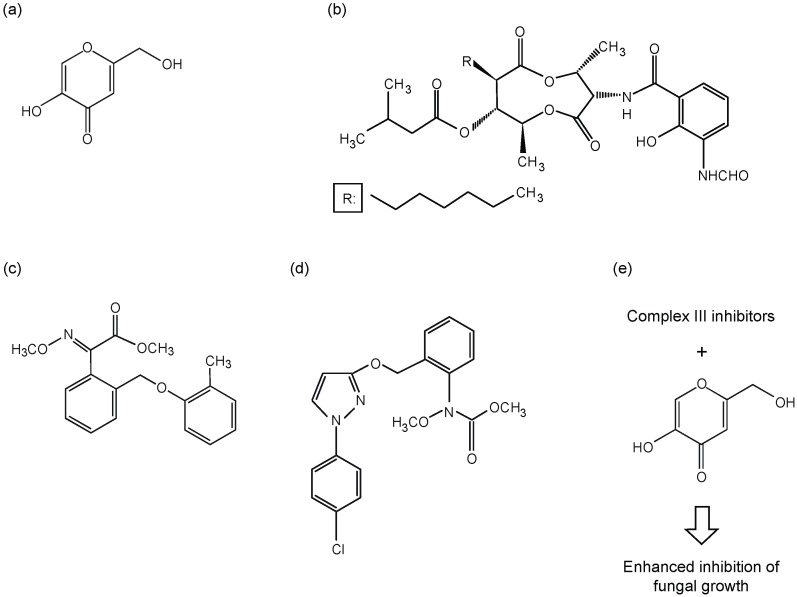
Structures of antifungal compounds examined in this study. (**a**) KA, (**b**) AntA, (**c**) Kre-Me, and (**d**) PCS; (**e**) Scheme for enhancement of antifungal activities of complex III inhibitors by KA-mediated chemosensitization.

Kojic acid (KA, Figure 2a) is a natural product of some filamentous fungi, mainly certain species of *Aspergillus* or *Penicillium*. KA is widely used as a depigmenting agent due to its ability to inhibit the activity of tyrosinase, a key enzyme responsible for melanogenesis in melanoma and melanocytes [17,18,19,20]. From a clinical perspective, KA can potentially inhibit pathogen infection since: (1) it enhances host immunity by stimulating phagocytosis, generating reactive oxygen species (ROS) in macrophages, and potentiating phytohemagglutinin-based proliferation of lymphocytes [21,22]; (2) KA or its structural derivatives directly exert antimicrobial activity against fungal/bacterial pathogens [23]. For instance, KA functions as an antifungal agent against *Cryptococcus neoformans* (cryptococcosis), where KA also inhibits melanin synthesis necessary for fungal infectivity [24].

We previously showed that KA could act as a chemosensitizing agent when co-applied with the polyene antifungal drug amphotericin B (AMB) or hydrogen peroxide (H_2_O_2_) against various filamentous fungal or yeast pathogens [25]. The mechanism of antifungal chemosensitization appeared to be modulation of the function of the antioxidant system in the fungus. Noteworthy is that the degree/efficacy of KA-mediated antifungal chemosensitization was related to the kinds of fungal strain and/or drug examined [25]. This tendency is similar to the “drug-chemosensitizer specificity” found in atovaquone-mediated chemosensitization (see above).

In this study, we further investigated if KA, as a chemosensitizer, could improve the activities of complex III inhibitors of MRC (*i.e*., AntA, Kre-Me, PCS; see Figure 2b–d for structures and 2e for scheme), and thus, possess potential as an active pharmaceutical/agroceutical ingredient, against various filamentous fungi. We included a number of human and plant pathogens, as well as model fungal strains, in our tests (see Table 1; Figure 2e). We observed that human fungal pathogens, *i.e*., *Aspergillus fumigatus*, *A. terreus*, *Acremonium* sp., and *Scedosporium* sp., were the most sensitive strains to KA-mediated chemosensitization to complex III inhibitors.

**Table 1:** Fungal strains used in this study.

Fungal strains		Strain characteristics	Source/Reference
***Aspergillus* (Human pathogens)**			
A. fumigatus *MYA-3626*		Aspergillosis, Reference clinical strain	ATCC ^a^
*A. fumigatus* AF293		Aspergillosis, Reference clinical strain	SCVMC ^b^
*A. fumigatus* AF10		Aspergillosis, Reference clinical strain	SCVMC ^b^
*A. fumigatus* 94-46		Aspergillosis, Clinical isolate	SCVMC ^b^
*A. fumigatus* 92-245		Aspergillosis, Clinical isolate	SCVMC ^b^
*A. terreus* UAB673		Aspergillosis, Clinical isolate	CDC ^c^
*A. terreus* UAB680		Aspergillosis, Clinical isolate	CDC ^c^
*A. terreus* UAB698		Aspergillosis, Clinical isolate	CDC ^c^
**Other filamentous fungi (Human pathogens)**			
*Acremonium* sp. CIMR 95-103		Clinical isolate	SCVMC ^b^
*Scedosporium* sp. CIMR 09-246		Clinical isolate	SCVMC ^b^
***Aspergillus* (Plant pathogens, *etc*.)**			
*A. flavus* 4212 ^g^		Kojic acid producer, Plant pathogen, Human pathogen (aspergillosis)	NRRL ^d^
*A. parasiticus* 2999		Kojic acid producer, Plant pathogen	NRRL ^d^
*A. oryzae* A815		Research strain (model)	FGSC ^e^
*A. niger* 326		Plant pathogen	NRRL ^d^
*A. ochraceous* 5175		Plant pathogen	NRRL ^d^
*A. nidulans* A4		Research strain (model)	FGSC ^e^
***Penicillium* (Plant pathogens, *etc*.)**			
*P. expansum* 974	Plant pathogen		NRRL ^d^
*P. expansum* W1	Plant pathogen		[ 26]
*P. expansum* FR2	Plant pathogen, Fludioxonil resistant (FLUD^R^) mutant derived from *P. expansum* W1		[ 26]
*P. expansum* W2	Plant pathogen		[ 26]
*P. expansum* FR3		Plant pathogen, FLUD^R^ mutant derived from *P. expansum* W2	[ 26]
P. chrysogenum *824*		Fleming’s penicillin-producing strain	NRRL ^d^
P. griseofulvum *2159*		Plant pathogen	NRRL ^d^
*P. griseofulvum* 2300		Plant pathogen	NRRL ^d^
P. digitatum *786*		Plant pathogen	NRRL ^d^
P. italicum *983*		Plant pathogen	NRRL ^d^
*P. glabrum* 766		Plant pathogen	NRRL ^d^
**Yeasts**			
*Saccharomyces cerevisiae* BY4741		Model yeast, Parental strain (*Mat* a *his3*∆*1 leu2*∆*0 met15*∆*0 ura3*∆*0*)	SGD ^f^
*S. cerevisiae* *yap1*		Transcription factor Yap1p mutant derived from BY4741	SGD ^f^
*S. cerevisiae sod2*		Mitochondrial superoxide dismutase (Mn-SOD) mutant derived from BY4741	SGD ^f^
*S. cerevisiae sod1*		Cytosolic superoxide dismutase (Cu,Zn-SOD) mutant derived from BY4741	SGD ^f^
*S. cerevisiae glr1*		Glutathione reductase (Glr1p) mutant derived from BY4741	SGD ^f^

*^a^* ATCC, American Type Culture Collection, Manassas, VA, USA. ^b^ SCVMC, Santa Clara Valley Medical Center, San Jose, CA, USA. ^c^ CDC, Centers for Disease Control and Prevention, Atlanta, GA, USA. ^d^ NRRL, National Center for Agricultural Utilization and Research, USDA-ARS, Peoria, IL, USA. ^e^ FGSC, Fungal Genetics Stock Center, Kansas City, MO, USA. ^f^ SGD, *Saccharomyces cerevisiae* Genome Database [27]. ^g^*A. flavus* infects both plants and humans.

## 2. Results and Discussion

### 2.1. Enhancing Antifungal Activity of H2O2 or Complex III Inhibitors with KA Against Aspergillus or Penicillium Strains: Agar Plate Bioassay

Hydrogen peroxide acts similarly to host-derived ROS, as a host defense response against infecting pathogens. For example, patients with chronic granulomatous disease (CGD) experience high susceptibility to invasive infections by *Aspergillus* [28]. The phagocytic immune cells of CGD patients cannot induce an oxidative burst because they lack NADPH oxidase, necessary to generate superoxides, the precursor to the antimicrobial ROS H_2_O_2_ [28]. Although the infecting fungi rely on their cellular antioxidant system for protection from host ROS, application of KA further enhances host immunity by stimulating phagocytosis and generation of ROS in macrophages (see Introduction) [21,22].

We previously examined KA-mediated chemosensitization to H_2_O_2_ and AMB [25]. Besides disrupting fungal plasma membranes, AMB also induces fungal oxidative damage [29,30,31,32] by stimulating ROS production [33]. Thus, we surmised that the effect of KA + AMB would be similar to KA + H_2_O_2_. However, unlike with KA + AMB, chemosensitization did not occur with KA + H_2_O_2_ in any of the yeast pathogens tested. We concluded that the effectiveness of KA-mediated chemosensitization was fungal strain- and/or drug-specific [25].

Since complex III inhibitors, like AMB, also trigger cellular oxidative stress in fungi (see Introduction), we also compared the effect of KA + complex III inhibitors with that of KA + H_2_O_2_ in this study.

#### 2.1.1. Filamentous Fungi

**Figure 3:**
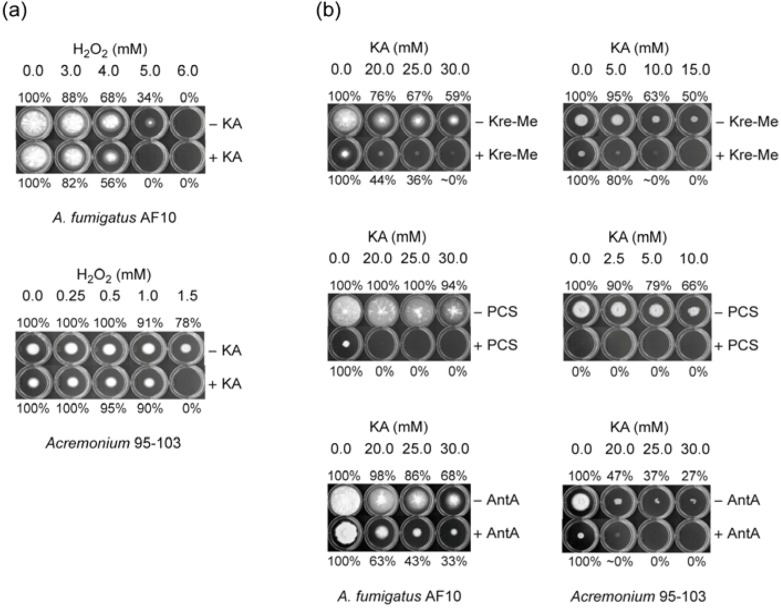
Exemplary agar (PDA) bioassays showing KA-mediated chemosensitization with (**a**) H_2_O_2_ or (**b**) complex III inhibitors, tested against *A. fumigatus* AF10 or *Acremonium* 95-103 (Note: No germination of *Acremonium* by PCS alone, reflecting hypersensitivity).

Our initial agar bioassays were performed with the human pathogenic fungi. Co-application of KA (5 mM) with H_2_O_2_ (Test concentrations: 0.0, 0.25, 0.5, 1.0, 1.5, 2.0, 2.5, 3.0, 3.5, 4.0, 5.0, 6.0, 7.0, 8.0, 9.0, 10.0 mM) resulted in increased antifungal activities of both compounds, compared to independent treatment of either KA or H_2_O_2_, alone (Figure 3a; Table 2). For example, co-application of H_2_O_2_ and KA at 5 mM, each, completely inhibited the growth of *A. fumigatus* AF10 (*i.e*., no visible germination on plates), whereas independent application of either H_2_O_2_ or KA, alone, did not achieve this level of antifungal activity. A similar level of chemosensitization was also observed in other fungi tested, *i.e*., *A. terreus*, *Acremonium*, and *Scedosporium*, by KA + H_2_O_2_ [Figure 3(a); see Table 2 for summary].

Next we found that KA-mediated chemosensitization could also be achieved with complex III inhibitors in most of the human pathogens tested (Figure 3b; Table 2). KA (Test concentrations: 0.0, 2.5, 5.0, 10.0, 15.0, 20.0, 25.0, 30.0 mM) was co-applied with 25 μM of complex III inhibitors (*i.e*., PCS, Kre-Me or AntA) in agar bioassays. For example, co-application of KA (20 mM or above) and PCS (25 μM) completely inhibited the growth of *A. fumigatus* AF10 (*i.e*., no visible germination on plates). Whereas, independent application of KA or PCS, alone, did not result in such a level of antifungal activity. Levels of enhancement of antifungal activity also depended upon types of complex III inhibitors co-applied. PCS exerted the highest activity, followed by Kre-Me and AntA. Similar trends were also observed in other pathogens, such as *A. terreus*, *Acremonium* and *Scedosporium*(Figure 3a; see Table 2 for summary). The only exceptions were *A. terreus* UAB698 (no enhancement of sensitivity by KA + any of the complex III inhibitors) and *A. terreus* UAB673/680 (no enhancement of sensitivity by KA + AntA), respectively. Therefore, sensitivity of fungal strains to KA-mediated chemosensitization with complex III inhibitors ranged, from highest to lowest, as follows: *Acremonium*, *Scedosporium* > *A. fumigatus* > *A. terreus*. Of note is that, although human pathogens were also sensitive to KA + H_2_O_2_, levels/degrees of their sensitivity were generally not parallel to that of KA + complex III inhibitors (see Table 2).

**Table 2:** Summary of responses of filamentous fungi to KA-mediated chemosensitization with H_2_O_2_ or complex III inhibitors (agar plate bioassay) ^a^.

Strains	H_2_O_2_	Kre-Me	PCS	AntA
Human pathogens				
*A. fumigatus* MYA-3626	++	+	++	+
*A. fumigatus* AF293	+ ^b^	+	++	+
*A. fumigatus* AF10	++	+	++	+
*A. fumigatus* 94-46	+	+	++	+
*A. fumigatus* 92-245	+	+	++	+
*A. terreus* UAB673	++ ^b^	+	++	-
*A. terreus* UAB680	+ ^b^	+	++	-
*A. terreus* UAB698	++ ^b^	-	-	-
*Acremonium* sp. 95-103	++	++	n/t ^c^	++
*Scedosporium* sp. 09-246	+	++	n/t ^c^	++
*Penicillium* strains				
*P. expansum* 974	++	-	-	-
*P. expansum* W1	+	-	-	-
*P. expansum* FR2	+	-	++	-
*P. expansum* W2	++	-	-	-
*P. expansum* FR3	++	-	+	-
*P. chrysogenum* 824	++	-	-	-
*P. griseofulvum* 2159	++	-	-	-
*P. griseofulvum* 2300	-	-	-	-
*P. digitatum* 786	++	n/t ^d^	n/t ^c^	+
*P. italicum* 983	-	+	++	-
*P. glabrum* 766	-	+	+	+
Other *Aspergillus* strains				
*A. flavus* 4212	-	-	-	-
*A. parasiticus* 2999	-	-	-	-
*A. oryzae* A815	-	-	-	-
*A. niger* 326	-	-	-	-
*A. ochraceous* 5175	-	-	-	-
*A. nidulans* A4	-	+	+	-

^a^ +, Enhancement of antifungal activity after co-application (reduced radial growth of fungi); ++, Enhancement of antifungal activity after co-application (no germination of fungi); -, No enhancement of antifungal activity after co-application. ^b^ [25]. ^c^ n/t, Not tested due to no growth of fungi w/ PCS (25 μM) alone (*i.e*., hypersensitivity to PCS alone). ^d^ n/t, Not tested due to no growth of fungi w/ Kre-Me (25 μM) alone (*i.e*., hypersensitivity to Kre-Me alone).

Agar bioassays performed on *Penicillium* strains, mostly plant pathogens, showed that co-application of KA with H_2_O_2_ resulted in enhancement of antifungal activities of both compounds (KA and H_2_O_2_), except *P. griseofulvum* 2300, *P. italicum* and *P. glabrum*, which were insensitive to this chemosensitization (Table 2; Figure data not shown).

KA-mediated chemosensitization was also performed using the complex III inhibitors on the *Penicillium* strains. Unlike the human pathogens tested, chemosensitization was more limited with the *Penicillium* strains, being effective only in strains, *P. expansum* FR2 and FR3 (both being fludioxonil (FLUD) resistant strains), *P. digitatum*, *P. italicum* and *P. glabrum* with KA + PCS (Table 2; PCS was the most effective complex III inhibitor in this test). Levels of strain sensitivity in decreasing order with KA + PCS were: *P. digitatum* > *P. italicum*, *P. expansum* FR2 > *P. glabrum*, *P. expansum* FR3. *P. digitatum*, *P. italicum*, and *P. glabrum* were also sensitive to KA + Kre-Me or AntA. However, *Penicillium* strains were generally not as sensitive to KA-mediated chemosensitization with complex III inhibitors as human pathogens. As observed in human pathogens, levels/degrees of fungal sensitivity to KA + H_2_O_2_ were not parallel to that of KA + complex III inhibitors (see Table 2).

Agar bioassays were performed on six other strains of *Aspergillus*, mainly plant pathogens or model strains (*A. flavus*: pathogenic to both plants and humans). These assays showed that co-application of KA with H_2_O_2_ or complex III inhibitors resulted in no enhancement of antifungal activity of any compound tested (KA, H_2_O_2_ or complex III inhibitors), except *A. nidulans*, which showed sensitivity to KA + PCS or Kre-Me (Table 2; Figure data not shown).

In summary, our results with filamentous fungi show that KA-mediated chemosensitization is fungal strain- or drug (compound)-dependent. Strain sensitivity to KA + complex III inhibitors and/or H_2_O_2_ varied as follows (in decreasing order): human pathogens (*A. fumigatus*, *A. terreus*, *Acremonium* sp., *Scedosporium* sp.; Mostly sensitive to KA + complex III inhibitors and KA + H_2_O_2_) > *Penicillium* species (Certain strains were sensitive to KA + complex III inhibitors, while many strains were sensitive to H_2_O_2_) > all other *Aspergillus* species (*A. flavus*, *A. parasiticus*, *A. oryzae*, *A. niger*, *A. ochraceous*, *A. nidulans*; Only *A. nidulans* was sensitive to KA +PCS or KA + Kre-Me. No strain was sensitive to KA + H_2_O_2_).

#### 2.1.2. Antioxidant, but Not Antifungal, Effect of KA in Wild Type and Antioxidant Mutants of the Model Yeast *Saccharomyces cerevisiae*

In our previous study, KA-mediated chemosensitization with H_2_O_2_ was not effective in any of the yeast pathogens tested [25]. Therefore, in the present study, we attempted to examine how the treatment of KA + H_2_O_2_ was related to various functions of antioxidant system of yeasts using *S. cerevisiae* as a model. For this study, we used a yeast dilution bioassay (see Experimental Section) and tested a wild type and four antioxidant mutant (gene knock-out) strains of *S. cerevisiae* as follows: (1) yap1Δ (Yap1p, a transcription factor, regulates the expression of four downstream genes within the antioxidant system, *i.e*., GLR1 (glutathione reductase), YCF1 (a glutathione S-conjugate pump), TRX2 (thioredoxin), and GSH1 (γ-glutamylcysteine synthetase [34,35]); (2) sod1Δ (Cu,Zn-SOD); (3) sod2Δ (Mn-SOD); and (4) glr1Δ (Glr1p, glutathione reductase; see Saccharomyces Genome Database [27]). These representative mutants play key roles in maintaining cellular redox homeostasis in both enzymatic (e.g., ROS radical-scavenging) and non-enzymatic (e.g., glutathione homeostasis) aspects. Worth noting is that *S. cerevisiae* has also been developed as a model system for studying atovaquone resistance [36].

**Figure 4:**
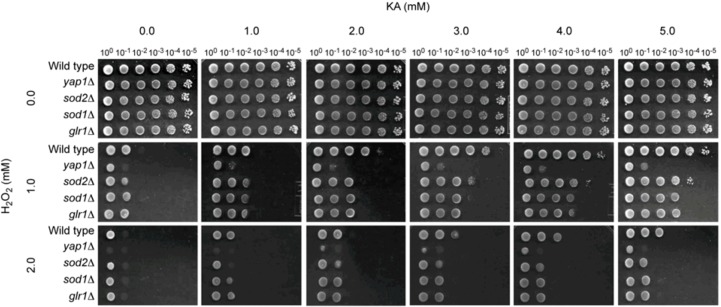
Agar (SG)-based yeast-cell dilution bioassay showing antioxidant effect of KA to H_2_O_2_-treated *S. cerevisiae* strains (10^0^ to 10^−5^: yeast dilution rates).

To our surprise, in these yeast strains, KA mainly acted as an antioxidant, but not as an antifungal chemosensitizer (Figure 4). For example, when wild type or mutants were treated with 1 mM of H_2_O_2_ alone, all yeast strains showed sensitive responses, as reflected in no growth of cells at 10^−2^ to 10^−5^ dilution spots (Figure 4). As expected, *yap1*Δ, which regulates the expression of four downstream genes in the antioxidant system, was more sensitive to H_2_O_2_ (*i.e*., no growth at 10^−1^ dilution spot) than any other yeast strains. However, as shown in Figure 4, co-application of KA with H_2_O_2_ ameliorated the H_2_O_2_-triggered oxidative stress, resulting in enhancement of the growth of all yeasts tested. For example, the wild type showed growth recovery up to 100,000-fold dilution (the 10^−5^ dilution spot), revealing this strain fully recovered from oxidative stress induced by H_2_O_2_ when KA was co-applied. Additionally, the sod1Δ, sod2Δ and glr1Δ mutants grew up to 10^−3^ to 10^−4^ dilution spots and yap1Δ grew up to 10^−1^ dilution spot when H_2_O_2_ was co-applied with 5 mM KA.

The antioxidant capacity of KA was also commensurate with KA concentrations. Although yeast strains showed increased sensitivity to 2 mM of H_2_O_2_, similar trends of antioxidation activity by KA were also observed (Figure 4). Thus, overall, the results indicate KA has a different effect, depending on types of fungi examined. That is, KA functions as an antioxidant in *S. cerevisiae*, while it acts as an antifungal chemosensitizer in certain of the filamentous fungi tested. KA may induce different transcriptional programs in *S. cerevisiae* than in filamentous fungi. Further studies, such as genome-wide gene expression profiling, are warranted to determine the precise mechanism of antioxidation in and/or insensitivity of yeast to KA + H_2_O_2_ chemosensitization.

### 2.2. Calculating Levels of Compound Interactions by Using Microtiter Plate (Microdilution) Bioassays: Human Pathogens, Penicillium Strains or *A. nidulans*

Based on results from the agar bioassay on filamentous fungi (shown above), levels of compound interactions between KA and PCS (the most potent complex III inhibitor according to this study) were assessed only for the strains sensitive to KA-mediated chemosensitization (*i.e*., most human pathogens, five *Penicillium* strains, and *A. nidulans*) using triplicate, microtiter-plate checkerboard bioassays (Clinical Laboratory Standards Institute (CLSI) M38-A) [37] with concentration ranges of KA (0, 1, 2, 4, 8, 16, 32, 64 mM) and PCS (0, 0.125, 0.25, 0.5, 1, 2, 4, 8, 16 µg/mL) (see Experimental Section). The effect of KA + Kre-Me (0, 0.125, 0.25, 0.5, 1, 2, 4, 8, 16 µg/mL) was also determined only for *Acremonium*, *Scedosporium* and *P. digitatum*, which were the most sensitive strains to complex III inhibitors (see Table 2).

#### 2.2.1. Co-Application of KA and PCS

Synergistic Fractional Inhibitory Concentration Indices (FICIs; see Experimental Section for calculations) were found between KA and PCS for most human pathogens (*A. fumigatus*, *A. terreus*, *Acremonium* sp., *Scedosporium* sp.) and *A. nidulans* (Table 3). Despite the absence of calculated “synergism”, as determined by “indifferent” interactions [38] (Table 3), there was enhanced antifungal activity of KA and PCS (*i.e*., chemosensitization) in *Acremonium*, which was reflected in lowered Minimum Inhibitory Concentrations (MICs) of each compound when combined. However, synergistic Fractional Fungicidal Concentration Indices (FFCIs) (at the level of ≥ 99.9% fungal death) between KA and PCS occurred only in *Acremonium* (Table 3), indicating the KA-mediated chemosensitization with PCS is fungistatic, not fungicidal, in most strains tested.

**Table 3:** Antifungal chemosensitization of KA (mM) to PCS (μg/mL) tested against filamentous fungi: summary of CLSI-based microdilution bioassays ^a^.

**Strains (Human pathogens and *A. nidulans*)**	**Compounds**	**MIC alone**	**MIC combined**	**FICI**
*A. fumigatus* AF293	KA	64	16	**0.3**
	PCS	>16 ^b^	1	
*A. fumigatus* MYA-3626	KA	>64 ^c^	16	**0.4**
	PCS	>16	8	
*A. fumigatus* AF10	KA	64	16	**0.4**
	PCS	>16	4	
*A. fumigatus* 92-245	KA	>64	16	**0.4**
	PCS	>16	8	
*A. fumigatus* 94-46	KA	>64	16	**0.4**
	PCS	>16	8	
*A. terreus* UAB673	KA	64	8	**0.1**
	PCS	>16	0.5	
*A. terreus* UAB 680	KA	64	8	**0.2**
	PCS	>16	1	
*A. nidulans* A4	KA	>64	32	**0.5**
	PCS	>16	8	
*Acremonium* sp. 95-103	KA	64	16	0.8
	PCS	0.25	0.125	
*Scedosporium* sp. 09-246	KA	64	4	**0.2**
	PCS	1	0.125	
Mean	KA	89.6	14.8	**0.3**
	PCS	25.7	3.9	
*t*-test ^d^	KA	-	*p* < 0.005	-
	PCS	-	*p* < 0.005	-
**Strains (Human pathogens and *A. nidulans*)**	**Compounds**	**MFC alone**	**MFC combined**	**FFCI**
*Acremonium* sp. 95-103	KA	>64	32	**0.5**
	PCS	2	0.5	
All other strains	KA	>64	>64	2
	PCS	>16	>16	
Mean	KA	128	118.4	1.9
	PCS	29	28.9	
*t*-test	KA	-	*p* < 0.5	-
	PCS	-	*p* < 1.0	-
**Strains (*Penicillium* strains)**	**Compounds**	**MIC alone**	**MIC combined**	**FICI**
*P. expansum* FR2	KA	>64	16	0.4
	PCS	2	0.5	
*P. expansum* FR3	KA	>64	32	0.8
	PCS	2	1	
*P. glabrum* 766	KA	>64	32	0.4
	PCS	>16	4	
*P. digitatum* 786	KA	>64	2	0.5
	PCS	0.25	0.125	
*P. italicum* 983	KA	>64	16	0.3
	PCS	>16	4	
Mean	KA	128	19.6	0.3
	PCS	13.7	1.9	
*t*-test	KA	-	*p* < 0.005	-
	PCS	-	*p* < 0.5	-
**Strains (*Penicillium* strains)**	**Compounds**	**MFC alone**	**MFC combined**	**FFCI**
*P. glabrum* 766	KA	>64	64	1
	PCS	>16	16	(99.8%)
*P. italicum* 983	KA	>64	64	1
	PCS	>16	16	(99.8%)
All other strains	KA	>64	> 64	2
	PCS	>16	>16	
Mean	KA	128	102.4	1.6
	PCS	32	25.6	
*t*-test	KA	-	*p* < 0.5	-
	PCS	-	*p* < 0.5	-

^a^ MIC: Minimum inhibitory concentration, MFC: Minimum fungicidal concentration, FICI: Fractional Inhibitory Concentration Indices, FFCI: Fractional Fungicidal Concentration Indices. Synergistic FICIs and FFCI were in bold. ^b^ PCS was tested up to 16 μg/mL. For calculation purpose, 32 μg/mL (doubling of 16 μg/mL) was used. ^c^ KA was tested up to 64 mM. For calculation purpose, 128 mM (doubling of 64 mM) was used. ^d^ Student’s *t*-test for paired data (combined, *i.e*., chemosensitization) was *vs*. mean MIC or MFC of each compound (alone, *i.e*., no chemosensitization) determined in strains (Calculation was based on [39]).

Synergistic FICIs between KA and PCS also occurred in four *Penicillium* strains (Table 3). Despite the absence of calculated “synergism” [38] (Table 3), there was enhanced antifungal activity of KA and PCS (*i.e*., chemosensitization) also in *P. expansum* FR3 (FLUD resistant strain), which was reflected in lowered MICs of each compound when combined. However, synergistic FFCI (at the level of ≥99.9% fungal death) between KA and PCS was not achieved in any of the *Penicillium* strains examined (Table 3), indicating that, as in the human pathogens/*A. nidulans* (See above), the KA-mediated chemosensitization with PCS is mostly fungistatic, not fungicidal, in *Penicillium* strains (Lowered Minimum Fungicidal Concentrations (MFCs), although not “synergistic” level, were observed in *P. glabrum* and *P. italicum* at the level of ≥99.8% fungal death; see Table 3).

#### 2.2.2. Strains Hypersensitive to Complex III Inhibitors: Testing *Acremonium*, *Scedosporium*, *P. digitatum* with Kre-Me

KA + Kre-Me was also examined in *Acremonium*, *Scedosporium* and *P. digitatum*, which were the most sensitive strains to complex III inhibitors (see Table 2). We tried to determine the level of sensitivity of these strains to Kre-Me, which is less potent than PCS (see Figure 3 and Table 2). Consistently, synergistic FICIs between KA and Kre-Me occurred in all strains tested (Table 4). However, synergistic FFCIs (at the level of ≥99.9% fungal death) between KA and Kre-Me were not achieved in any of the strains examined (Table 4), while lowered MFCs for both KA and Kre-Me were observed in *Acremonium* (FFCI = 0.6). *Acremonium* sp. is the only strain with low FFCI values for both PCS and Kre-Me, *i.e*., 0.5_PCS_ and 0.6_Kre-Me_, respectively (see Table 3, Table 4). Results further confirmed the sensitive responses of *Acremonium*, *Scedosporium* and *P. digitatum* to complex III inhibitors (both PCS and Kre-Me).

**Table 4:** Antifungal chemosensitization of KA (mM) to Kre-Me (μg/mL) tested against *Acremonium*, *Scedosporium* or *P. digitatum* strains: summary of CLSI-based microdilution bioassays ^a^.

**Strains**	**Compounds**	**MIC alone**	**MIC combined**	**FICI**
*Acremonium* sp. 95-103	KA	64	8	**0.2**
	Kre-Me	16	1	
*Scedosporium* sp. 09-246	KA	64	8	**0.2**
	Kre-Me	>16 ^b^	1	
*P. digitatum* 786	KA	>64 ^c^	8	**0.1**
	Kre-Me	8	0.5	
Mean	KA	85.3	8	**0.1**
	Kre-Me	18.7	0.8	
*t*-test ^d^	KA	-	*p* < 0.05	-
	Kre-Me	-	*p* < 0.1	-
**Strains**	**Compounds**	**MFC alone**	**MFC combined**	**FFCI**
*Acremonium* sp. 95-103	KA	>64	64	0.6
	Kre-Me	>16	2	
*Scedosporium* sp. 09-246 & *P. digitatum* 786	KA	>64	>64	2
	Kre-Me	>16	>16	
Mean	KA	128	106.7	1.5
	Kre-Me	32	22	
*t*-test ^d^	KA	-	*p* < 0.5	-
	Kre-Me	-	*p* < 0.5	-

^a^ MIC: Minimum inhibitory concentration, MFC: Minimum fungicidal concentration, FICI: Fractional Inhibitory Concentration Indices, FFCI: Fractional Fungicidal Concentration Indices. Synergistic FICIs were in bold. ^b^ Kre-Me was tested up to 16 μg/mL. For calculation purpose, 32 μg/mL (doubling of 16 μg/mL) was used. ^c^ KA was tested up to 64 mM. For calculation purpose, 128 mM (doubling of 64 mM) was used. ^d^ Student’s *t*-test for paired data (combined, *i.e*., chemosensitization) was *vs*. mean MIC or MFC of each compound (alone, *i.e*., no chemosensitization) determined in three strains (Calculation was based on [39]).

The results of all CLSI-based checkerboard (chemosensitization) tests (*i.e*., KA + PCS or Kre-Me in filamentous fungi) are summarized in Table 5. As shown in the Table, the FICIs for thirteen strains (out of fifteen strains) w/PCS and for three fungi (the most sensitive strains to complex III inhibitors) w/Kre-Me were synergistic. Whereas, FFCI for only *Acremonium* sp. was synergistic, indicating the KA-mediated chemosensitization with complex III inhibitors exerted mostly fungistatic (but not fungicidal) effects.

**Table 5:** Summary of responses of filamentous fungi to the co-application of KA with PCS and/or Kre-Me (CLSI-based microdilution bioassays).

Fungal strains	Agents co-applied	
	PCS (FICI, FFCI) ^a^	Kre-Me (FICI, FFCI) ^a^
**Human pathogens**		
*A. fumigatus* AF293	(**0.3**, 2.0)	nt ^b^
*A. fumigatus* MYA-3626	(**0.4**, 2.0)	nt
*A. fumigatus* AF10	(**0.4**, 2.0)	nt
*A. fumigatus* 92-245	(**0.4**, 2.0)	nt
*A. fumigatus* 94-46	(**0.4**, 2.0)	nt
*A. terreus* UAB673	(**0.1**, 2.0)	nt
*A. terreus* UAB680	(**0.2**, 2.0)	nt
*Acremonium* sp. 95-103	(**0.8**, **0.5**)	(**0.2**, 0.6)
*Scedosporium* sp. 09-246	(**0.2**, 2.0)	(**0.2**, 2.0)
**Plant pathogens**		
*P. expansum* FR2	(**0.4**, 2.0)	nt
*P. expansum* FR3	(0.8, 2.0)	nt
*P. glabrum* 766	(**0.4**, 1.0)	nt
*P. italicum* 983	(**0.3**, 1.0)	nt
*P. digitatum* 786	(**0.5**, 2.0)	(**0.1**, 2.0)
**Other *Aspergillus***		
*A. nidulans* A4	(**0.5**, 2.0)	nt

^a^ FICI, Fractional Inhibitory Concentration Indices; FFCI, Fractional Fungicidal Concentration Indices; Both FICI and FFCI values were based on Table 3, Table 4; Bold: synergistic interaction. ^b^ nt, not tested.

## 3. Experimental

### 3.1. Fungal Strains and Culture Conditions

Human pathogens (*Aspergillus fumigatus*, *A. terreus*, *Acremonium* sp., *Scedosporium* sp.) (see Table 1) were grown at 35 °C on potato dextrose agar (PDA; Sigma, St. Louis, MO, USA). All other filamentous fungi were grown at 28 °C on PDA. Yeast strains (*i.e*., wild type and gene deletion mutants of *Saccharomyces cerevisiae*; see Table 1) were cultured on Synthetic Glucose (SG; Yeast nitrogen base without amino acids 0.67%, glucose 2% with appropriate supplements: uracil 0.02 mg/mL, amino acids 0.03 mg/mL) or Yeast Peptone Dextrose (YPD; Bacto yeast extract 1%, Bacto peptone 2%, glucose 2%) medium at 30 °C.

### 3.2. Chemicals

Antifungal chemosensitizing agent [kojic acid (KA)], antifungal drugs [antimycin A (AntA), kresoxim methyl (Kre-Me), pyraclostrobin (PCS)] and oxidizing agent [hydrogen peroxide (H_2_O_2_)] were procured from Sigma Co. Each compound was dissolved in dimethyl sulfoxide (DMSO; absolute DMSO amount: <1% in media), except H_2_O_2_, which was dissolved in water, before incorporation into culture media. In all tests, control plates (*i.e*., “No treatment”) contained DMSO at levels equivalent to that of cohorts receiving antifungal agents, within the same set of experiments.

### 3.3. Antifungal Bioassay

#### 3.3.1. Agar Plate Bioassay: Filamentous Fungi

In the agar plate bioassay, measurement of sensitivities of filamentous fungi to the antifungal agents was based on percent (%) radial growth of treated compared to control (“No treatment”) fungal colonies (see text for test concentrations.) [40]. Minimum inhibitory concentration (MIC) values on agar plates were determined based on triplicate bioassays, and defined as the lowest concentration of agents where no fungal growth was visible on the plate. For the above assays, fungal conidia (5 × 10^4^ CFU/mL) were diluted in phosphate-buffered saline (PBS) and applied as a drop onto the center of PDA plates with or without antifungal compounds. Growth was observed for three to seven days to determine cellular sensitivities to compounds.

#### 3.3.2. Microtiter Plate (microdilution) Bioassay: Filamentous Fungi

To determine antifungal chemosensitizing activities of KA (0, 1, 2, 4, 8, 16, 32, 64 mM) to complex III inhibitors (PCS or Kre-Me: 0, 0.125, 0.25, 0.5, 1, 2, 4, 8, 16 µg/mL) in filamentous fungi, checkerboard bioassays (0.4 × 10^4^–5 × 10^4^ CFU/mL) were performed in microtiter wells using a broth microdilution method (with RPMI 1640 medium; Sigma Co.), according to those outlined by the Clinical Laboratory Standards Institute (CLSI) M38-A [37]. Minimum inhibitory concentrations (MICs), lowest concentration of agent(s) showing no visible fungal growth, were assessed after 48 and 72 h. Minimum fungicidal concentrations (MFCs), lowest concentration of agents showing ≥ 99.9% fungal death (except where noted in Tables), were determined (following completion of MIC assays) wherein entire volumes of microtiter wells (200 μL) were spread onto individual PDA plates, and cultured for another 48 and 72 h. Compound interactions, Fractional Inhibitory Concentration Indices (FICIs) and Fractional Fungicidal Concentration Indices (FFCI), were calculated as follows: FICI or FFCI = (MIC or MFC of compound A in combination with compound B/MIC or MFC of compound A, alone) + (MIC or MFC of compound B in combination with compound A/MIC or MFC of compound B, alone). Interactions were defined as: “synergistic” (FICI or FFCI ≤ 0.5) or “indifferent” (FICI or FFCI > 0.5–4) [38]. Statistical analysis was based on [39].

#### 3.3.3. Agar Plate Bioassay: *S. cerevisiae*

Petri plate-based yeast dilution bioassays were performed on the wild type and antioxidant mutants (*yap1*Δ, sod1Δ, sod2Δ, glr1Δ) to assess effects of KA + H_2_O_2_ on the antioxidant system. Yeast strains were exposed to 1 to 5 mM of KA, w/o or w/H_2_O_2_ (1 or 2 mM) on SG for 5 to 7 days. These assays were performed in duplicate on SG agar following previously described protocols [41].

## 4. Conclusions

In this study, KA enhanced antifungal activities of MRC inhibitor(s) or H_2_O_2_ as follows: (1) Most human pathogens tested (*i.e*., *A. fumigatus*, *A. terreus*, *Acremonium* sp., *Scedosporium* sp.) were sensitive to both KA + complex III inhibitors and KA + H_2_O_2_, except *A. terreus* UAB698 (no chemosensitization w/all complex III inhibitors tested) and *A. terreus* UAB673/680 (no chemosensitization w/AntA); (2) Most of the plant pathogenic *Penicillium* species were sensitive to KA + H_2_O_2_, except *P. griseofulvum* 2300, *P. italicum* and *P. glabrum* (no chemosensitization); (3) Some *Penicillium* species (*i.e*., *P. digitatum*, *P. italicum*, *P. glabrum*, and FLUD-resistant *P. expansum* FR2/FR3) were sensitive to KA + at least one of the complex III inhibitors. However, all other *Penicillium* species were insensitive to KA + complex III inhibitors (no chemosensitization); (4) All other *Aspergillus* species (*i.e*., *A. flavus*, *A. parasiticus*, *A. oryzae*, *A. niger*, *A. ochraceous*, *A. nidulans*) were insensitive to KA + complex III inhibitors and/or KA + H_2_O_2_ (no chemosensitization), except *A. nidulans*, which was sensitive to KA + PCS or Kre-Me (chemosensitization). Further studies are required to determine the mechanism(s) governing the variability of these *Aspergillus* strains to KA-mediated chemosensitization; (5) Most compound interactions at MIC level (*i.e*., FICI) between KA and PCS or Kre-Me, determined by CLSI method, resulted in synergism, except *Acremonium* sp. (KA + PCS) and *P. expansum* FR3 (KA + Kre-Me), which resulted in a certain level of positive interaction between compounds, but not synergism; (6) The antifungal chemosensitizing capacity of KA appears to be fungal strain-specific (*i.e*., specific for certain human pathogens or *Penicillium* species only) as well as fungal isolate-dependent (*i.e*., *A. terreus*). KA mainly functions as an antioxidant in yeasts; and (7) Strain sensitivity to KA + complex III inhibitors or H_2_O_2_ varied as follows (in decreasing order): human fungal pathogens > *Penicillium* species > all other *Aspergillus* species.

In conclusion, KA, which is a relatively safe, natural compound to humans [42], shows some potential to serve as an antifungal chemosensitizing agent in combination with complex III inhibitors. This potential appears to be greatest with those filamentous fungi tested that are mainly pathogenic to humans. Chemosensitization can lower dosage levels of antifungal drugs necessary for effective control of fungi. Thus, use of safe chemosensitizing agents that selectively debilitate the fungal pathogen may be a viable approach to circumvent potential side-effects commonly associated with antimycotic therapy.
